# Discordance in the diagnostic assessment of vulnerable plaques between radiofrequency intravascular ultrasound versus optical coherence tomography among patients with acute myocardial infarction: insights from the IBIS-4 study

**DOI:** 10.1007/s10554-021-02272-6

**Published:** 2021-07-08

**Authors:** Yasushi Ueki, Kyohei Yamaji, Sylvain Losdat, Alexios Karagiannis, Masanori Taniwaki, Marco Roffi, Tatsuhiko Otsuka, Konstantinos C. Koskinas, Lene Holmvang, Rafaela Maldonado, Giovanni Pedrazzini, Maria D. Radu, Jouke Dijkstra, Stephan Windecker, Hector M. Garcia-Garcia, Lorenz Räber

**Affiliations:** 1 Department of Cardiology, Inselspital, Bern University Hospital, University of Bern, 3010 Bern, Switzerland; 2 Division of Cardiology, Kokura Memorial Hospital, Kitakyushu, Japan; 3 CTU Bern, University of Bern, Bern, Switzerland; 4 Department of Cardiology, Tokorozawa Heart Center, Tokorozawa, Japan; 5 Division of Cardiology, University Hospital Geneva, Geneva, Switzerland; 6 Heart Center, Rigshospitalet, Copenhagen University Hospital, Copenhagen, Denmark; 7 Cardiocentro, Lugano, Switzerland; 8 Leiden University Medical Center, Leiden, the Netherlands; 9 MedStar Cardiovacular Research Network, MedStar Washington Hospital Center, Washington, DC USA

**Keywords:** Fibroatheroma, Intravascular ultrasound, Optical coherence tomography, Radiofrequency

## Abstract

We aimed to evaluate the diagnostic agreement between radiofrequency (RF) intravascular ultrasound (IVUS) and optical coherence tomography (OCT) for thin-cap fibroatheroma (TCFA) in non-infarct-related coronary arteries (non-IRA) in patients with ST-segment elevation myocardial infarction (STEMI). In the Integrated Biomarker Imaging Study (IBIS-4), 103 STEMI patients underwent OCT and RF-IVUS imaging of non-IRA after successful primary percutaneous coronary intervention and at 13-month follow-up. A coronary lesion was defined as a segment with ≥ 3 consecutive frames (≈1.2 mm) with plaque burden ≥ 40% as assessed by grayscale IVUS. RF-IVUS-derived TCFA was defined as a lesion with > 10% confluent necrotic core abutting to the lumen in > 10% of the circumference. OCT-TCFA was defined by a minimum cap thickness < 65 μm. The two modalities were matched based on anatomical landmarks using a dedicated matching software. Using grayscale IVUS, we identified 276 lesions at baseline (N = 146) and follow-up (N = 130). Using RF-IVUS, 208 lesions (75.4%) were classified as TCFA. Among them, OCT identified 14 (6.7%) TCFA, 60 (28.8%) thick-cap fibroatheroma (ThCFA), and 134 (64.4%) non-fibroatheroma. All OCT-TCFA (n = 14) were confirmed as RF-TCFA. The concordance rate between RF-IVUS and OCT for TCFA diagnosis was 29.7%. The reasons for discordance were: OCT-ThCFA (25.8%); OCT-fibrous plaque (34.0%); attenuation due to calcium (23.2%); attenuation due to macrophage (10.3%); no significant attenuation (6.7%). There was a notable discordance in the diagnostic assessment of TCFA between RF-IVUS and OCT. The majority of RF-derived TCFA were not categorized as fibroatheroma using OCT, while all OCT-TCFA were classified as TCFA by RF-IVUS.

**ClinicalTrials.gov Identifier** NCT00962416.

**Supplementary Information:**

The online version contains supplementary material available at 10.1007/s10554-021-02272-6.

## Introduction

Acute coronary syndrome is commonly caused by plaque rupture which results in luminal thrombosis. Thin-cap fibroatheroma (TCFA), a precursor lesion for plaque rupture, is characterized by a large necrotic core with an overlaying thin fibrous cap [1]. Radiofrequency (RF) intravascular ultrasound (IVUS) and optical coherence tomography (OCT) represent two intracoronary imaging techniques used for in-vivo TCFA detection, although each applies distinct methodologies. RF-IVUS involves spectral analysis of radiofrequency backscatter data to construct tissue maps according to the classification tree and classify plaque into four major components such as necrotic core (NC), dense calcium, fibrous, and fibrofatty tissue [2]. RF-TCFA is diagnosed based on the visual assessment of these outputs according to the amount and location of NC [3]. OCT is an imaging technique using near infrared light offering a detailed visual plaque characterization with a high-resolution image. Digital images generated using the echo time delay of the emitted light and the intensity of the received light are visually assessed to diagnose OCT-TCFA based on angle and fibrous cap thickness of fibroatheroma (i.e. < 65 μm) [4]. Previous clinical studies have demonstrated that the presence of TCFA defined either by RF-IVUS or OCT was associated with future major adverse cardiovascular events [5–8].

Although both techniques have been validated histologically to a certain degree for necrotic core/lipid pool [9–11], to date, appropriately sized in vivo imaging studies comparing the diagnostic agreement between RF-IVUS and OCT for TCFA are lacking. We, therefore, aimed to evaluate the diagnostic agreement between RF-IVUS and OCT for the assessment of TCFA in non-infarct-related coronary arteries (non-IRA) in patients presenting with ST-segment elevation myocardial infarction (STEMI).

## Methods

The integrated biomarker imaging study (IBIS-4) was a prospective cohort study nested into the COMFORTABLE-AMI, which compared biolimus-eluting stents with bare metal stents in 1161 STEMI patients undergoing primary percutaneous coronary intervention (PCI) at 11 international centers [12–14]. A total of 103 patients were enrolled in the IBIS-4 at five pre-defined centers using serial (i.e. baseline and 13 months) RF-IVUS and OCT to quantify changes in atherosclerotic plaque characteristics in non-IRA under long-term high-intensity statin therapy (rosuvastatin of 20 mg during the first 2 weeks, followed by 40 mg). For the present study, patients not available for (1) paired pullbacks (i.e. OCT and RF-IVUS) per vessel or (2) matched OCT-analyzed segments with IVUS-defined lesions were excluded. All patients provided written informed consent and the study was approved by the institutional review boards of all participating centers.

### Study procedure

The details of image acquisition and analysis methodology have been published previously [14, 15]. In brief, following successful primary PCI (i.e. TIMI flow ≥ 2), the proximal 50 mm of two non-infarct related arteries were imaged by IVUS, RF-IVUS, and OCT. The region of interest was selected between two anatomical landmarks (distal: sidebranch, proximal: LM bifurcation or ostium of the RCA). Grayscale IVUS and RF-IVUS images were acquired with a 20-MHz ultrasound catheter (Eagle Eye, s5, Volcano Cooperation, Rancho Cordova, CA) and OCT images with the commercially available Fourier-domain C7-XR imaging system using the Dragonfly™ imaging catheter (St. Jude Medical, St. Paul, MN, USA). Patients were scheduled for repeat intracoronary imaging at 13 months.

### IVUS and OCT analysis

Definitions of IVUS and OCT variables have been published previously [14, 15]. Offline grayscale IVUS and RF-IVUS analysis were performed with QIVUS software (Medis, Leiden, The Netherlands) at the independent core laboratory (Cardialysis, B.V., Rotterdam, The Netherlands). The lumen and the external elastic membrane were measured in every ≈ 0.4 mm. A lesion was defined as ≥ 3 consecutive frames (≈ 1.2 mm) with plaque burden ≥ 40% as assessed by grayscale IVUS and considered separate when the distance between adjacent lesions was more than 5 mm. RF-IVUS-derived lesion type including TCFA, thick cap fibroatheroma (ThCFA), fibrocalcific, fibrotic, and pathological intimal thickening was determined by an independent Corelab (Cardialysis, Rotterdam) under the guidance of a senior cardiologist (H.G.) according to the consensus methodology [3]. RF-TCFA was defined as a lesion with > 10% confluent NC abutting to the lumen in > 10% of the circumference in ≥ 3 consecutive frames [3, 16]. Offline OCT analysis was performed every 0.4 mm using QCU-CMS V 4.69 (Medis, Leiden, The Netherlands) at the Bern University core laboratory. Plaque classification by means of OCT was diagnosed by analysts blinded to RF-IVUS (K.Y., R.M., L.R.). Fibroatheroma as assessed by OCT was defined as plaque with evidence of lipid pool > 90° and no lateral delineation. TCFA were defined by a minimum FCT ≤ 65 µm, whereas thick-cap fibroatheromas (ThCFA) were fibroatheromas with minimum FCT > 65 µm. The principal reasons for the discordance between RF-TCFA and OCT-non-TCFA were qualitatively assessed by two analysts (Y.U. and K.Y.) (and the third referee [L.R.] in case of disagreement). Pre-defined reasons included OCT-ThCFA; OCT-fibrous plaque; attenuation due to calcium; attenuation due to macrophage; no significant attenuation. The two modalities were matched based on anatomical landmarks using a dedicated matching software (IvusOctRegistration, Version 16, Medis, Leiden, The Netherlands). Remodeling index was calculated as external elastic membrane (EEM) area of the frame with the smallest lumen area within the lesion divided by mean EEM area of frames with minimum plaque burden within the proximal and distal 5-mm regions adjacent to the lesion [17] (Tables 1, 2).

**Table 1:** Patient characteristics

Variables	N = 88
Age (years)	58.7 ± 10.1
Male sex	81 (82.0%)
Body mass index (m^2^/kg)	27.7 ± 4.0
Hypertension	41 (46.6%)
Diabetes	11 (12.5%)
Dyslipidemia	36 (40.9%)
Current smoker	36 (40.9%)
Family history of coronary artery disease	25 (29.4%)
Renal failure (eGFR < 60 ml/min/1.73m^2^)	5 (5.7%)
Previous myocardial infarction	2 (2.3%)
Previous percutaneous coronary intervention	1 (1.1%)
Medication at discharge	
Aspirin	87 (100%)
Prasugrel	69 (79.3%)
Clopidogrel	18 (20.7%)
Any DAPT	87 (100%)
Statin	87 (100%)

Values are n (%) or mean ± standard deviations

*DAPT* dual antiplatelet therapy, *eGFR* estimated glomerular filtration rate

**Table 2:** Plaque classification as assessed by RF-IVUS and OCT

Lesions (n = 276)					
RF-IVUS	OCT				
	TCFA (n = 14)	ThCFA (n = 77)	Fibrocalcific plaque (n = 126)	Fibrous plaque (n = 47)	Normal vessel (n = 12)
TCFA (n = 208)	14	60	101	29	4
ThCFA (n = 39)	0	12	19	7	1
Fibrocalcific (n = 3)	0	0	3	0	0
Fibrotic (n = 9)	0	2	1	4	2
PIT (n = 17)	0	3	2	7	5

*IVUS* intravascular ultrasound, *OCT* optical coherence tomography, *PIT* pathological intimal thickening, *TCFA* thin-cap fibroatheroma, *ThCFA* thick-cap fibroatheroma, *RF* radiofrequency

### Statistical analysis

Categorical variables are shown as absolute values and percentages and were analyzed using Fisher’s exact tests. Continuous variables are expressed as mean ± standard deviation and were analyzed using Student’s *t* tests. To compare IVUS and OCT parameters according to the concordance between RF- and OCT-TCFA (Table 3), we ran mixed-effect models in which lesion identity nested within patient identity were fitted as random intercept to account for the non-independence of (i) lesions being measured twice (at baseline and follow-up) and (ii) lesions originating from the same patient. P values were two-tailed and the significance level was set to 0.05 in all analyses. Statistical analyses were performed using Stata 15 (StataCorp, College Station, TX, USA).

**Table 3:** IVUS and OCT analysis according to the concordance between RF- and OCT-TCFA

	RF-TCFA and OCT-TCFA (n = 14)	RF-TCFA and OCT-non-TCFA (n = 194)	RF-non-TCFA and OCT-non-TCFA (n = 68)	P value
IVUS				
Average vessel area (mm^2^)	16.4 ± 4.4	17.3 ± 5.6	14.3 ± 4.9	0.273
Average lumen area (mm^2^)	7.1 ± 2.0	8.7 ± 3.0	7.6 ± 2.7	0.018
Minimum lumen area (mm^2^)	4.0 ± 2.0	6.0 ± 2.7	5.9 ± 2.8	0.545
Percent atheroma volume (%)	56.9 ± 4.7	50.2 ± 6.0	47.0 ± 5.9	< 0.001
Total atheroma volume (mm^3^)	287 ± 120	194 ± 136	107 ± 136	0.001
Remodeling index	1.23 ± 0.79	1.02 ± 0.31	0.98 ± 0.19	0.001
RF-IVUS				
Percent fibrous area (%)	54.6 ± 7.5	56.7 ± 8.7	64.3 ± 9.7	< 0.001
Percent fibrofatty area (%)	8.8 ± 5.2	10.3 ± 5.6	13.9 ± 6.4	< 0.001
Percent necrotic core area (%)	27.1 ± 7.7	23.3 ± 6.5	16.4 ± 7.7	< 0.001
Percent dense calcium area (%)	9.5 ± 4.4	9.6 ± 6.3	5.4 ± 6.3	< 0.001
OCT				
Average lumen area (mm^2^)	6.9 ± 2.6	8.6 ± 3.5	7.7 ± 3.1	0.197
Minimum lumen area (mm^2^)	3.9 ± 2.3	5.9 ± 3.2	6.0 ± 3.4	0.582
Percent of frames with TCFA (%)	10.3 ± 7.5	0	0	< 0.001
Percent of frames with ThCFA (%)	46.1 ± 22.0	14.4 ± 27.7	14.0 ± 27.0	0.025
Percent of frames with fibrous plaque (%)	30.3 ± 20.5	44.6 ± 32.6	44.0 ± 36.4	0.194
Percent of frames with fibrocalcific plaque (%)	12.9 ± 13.5	33.1 ± 32.5	20.8 ± 31.1	0.373
Percent of frames with normal vessel (%)	0.5 ± 1.0	7.9 ± 19.0	21.2 ± 34.8	0.134
Percent of frames with macrophage lines (%)	25.5 ± 17.7	17.6 ± 20.2	10.3 ± 16.3	0.051
Mean macrophage angle (°)	13.9 ± 16.1	10.6 ± 18.3	5.4 ± 11.5	0.302
Fibroatheroma				
Minimum cap thickness (um)	46.7 (8.9)	133 (77.1)	120 (34.4)	< 0.001
Mean cap thickness (um)	223 (46.8)	345 (106)	350 (88.1)	< 0.001
Mean lipid arc (°)	177 (41.8)	133 (38.6)	130 (31.2)	< 0.001

Values are mean ± standard deviations

*IVUS* intravascular ultrasound, *OCT* optical coherence tomography, *TCFA* thin-cap fibroatheroma, *RF* radiofrequency

## Results

### Patient population

**Fig. 1:**
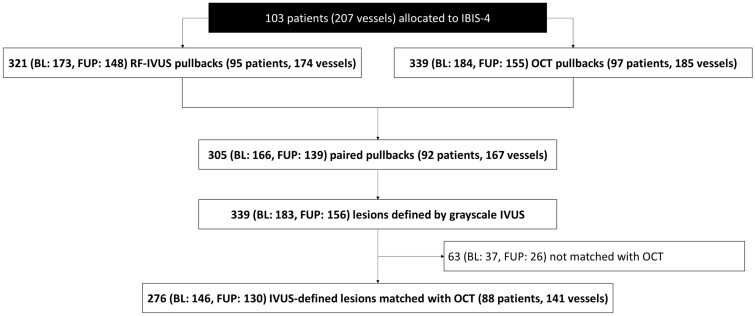
Patient flow. *BL* baseline, *FUP* follow-up, *IVUS* intravascular ultrasound, *OCT* optical coherence tomography, *STEMI* ST-elevation myocardial infarction, *RF* radiofrequency

Among 103 patients enrolled into IBIS-4, 276 IVUS-defined lesions matched with OCT (baseline: 146, follow-up: 130) were available in 88 patients/141 vessels (Fig. 1). Patient characteristics are summarized in Table 1. Mean age was 59 ± 10 years, 82% were male, and 13% had diabetes mellitus.

### Fibroatheroma as assessed by RF-IVUS and OCT

**Fig. 2:**
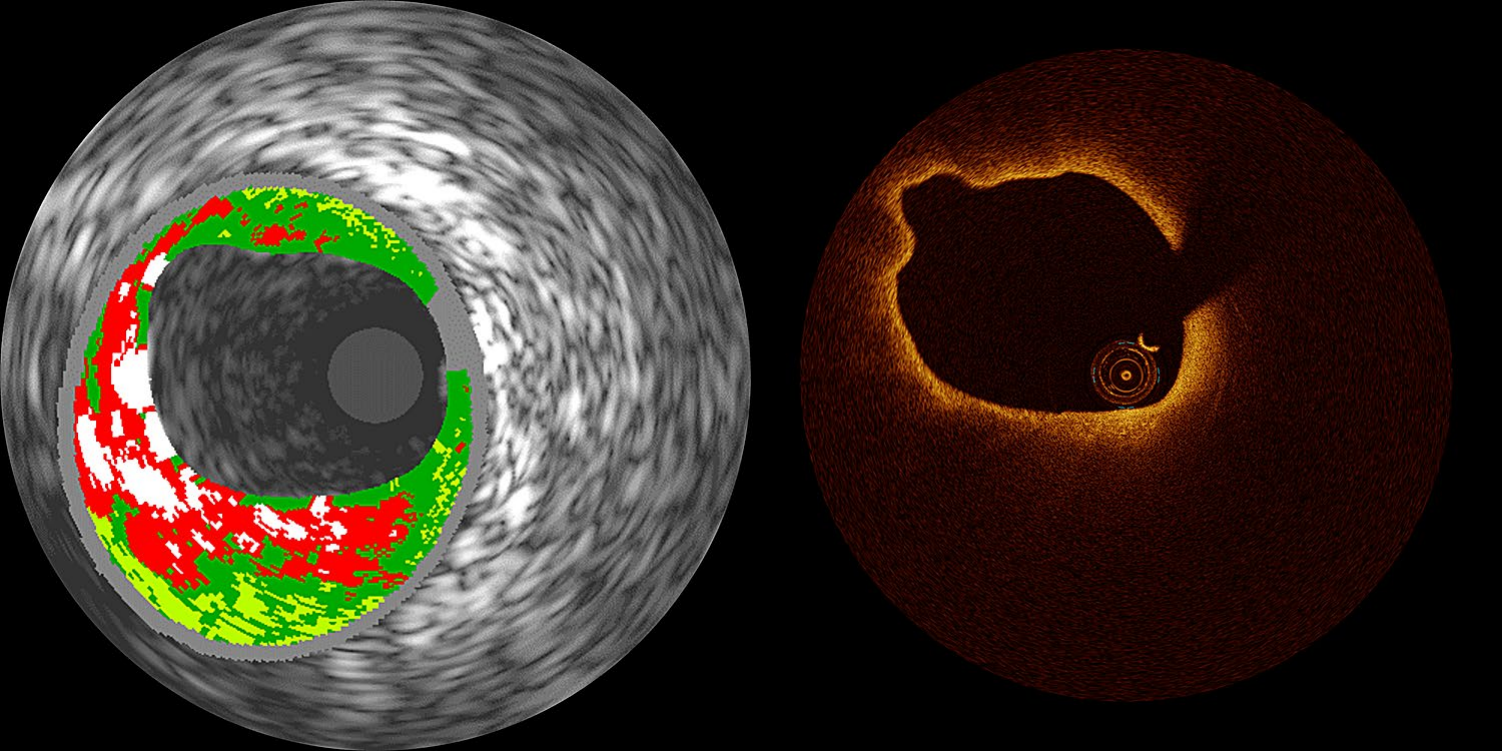
Representative case of TCFA determined by RF-IVUS and OCT

**Fig. 3:**
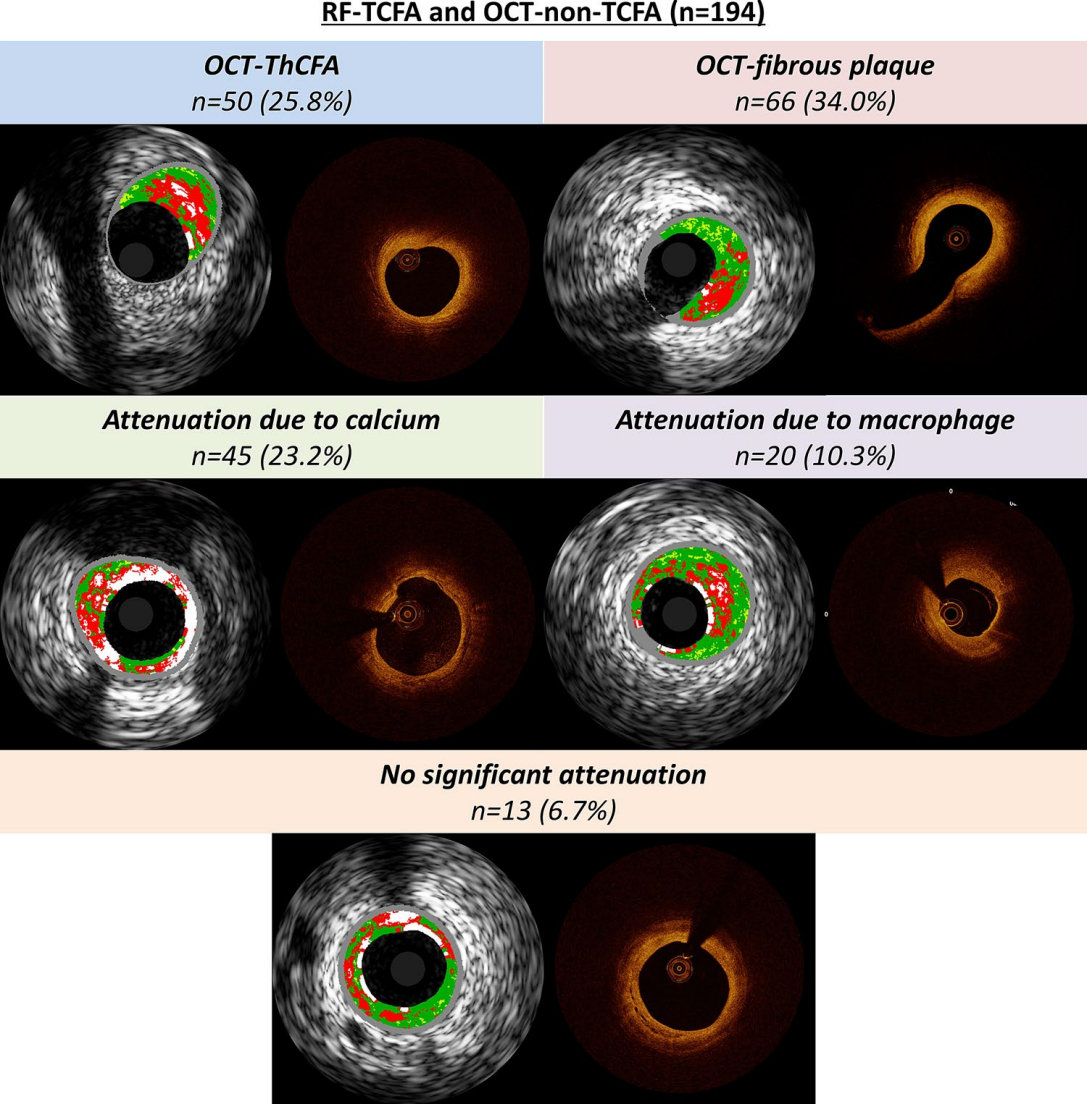
Principal reasons for discordance between RF-IVUS and OCT. *IVUS* intravascular ultrasound, *OCT* optical coherence tomography, *TCFA* thin-cap fibroatheroma, *ThCFA* thick-cap fibroatheroma, *RF* radiofrequency

Table 2 summarizes plaque classifications as assessed by RF-IVUS and OCT. Using RF-IVUS, 208 lesions (75.4%) were classified as TCFA. Among them, 14 (6.7%) RF-TCFA were classified as TCFA by OCT, 60 (28.8%) as ThCFA, 101 (48.6%) as fibrocalcific, 29 (13.9%) as fibrous, and 4 (1.9%) as normal vessel. All OCT-TCFA (n = 14) were confirmed as RF-TCFA (Fig. 2). The concordance rate between RF-IVUS and OCT for TCFA diagnosis was 29.7% (RF-TCFA and OCT-TCFA: 5.1%, RF-non-TCFA and OCT-non-TCFA: 24.6%). There were 194 lesions (70.3%) classified as RF-TCFA but OCT-non-TCFA. The reasons for discordance were: OCT-ThCFA (n = 50, 25.8%); OCT-fibrous plaque (n = 66, 34.0%); attenuation due to calcium (n = 45, 23.2%); attenuation due to macrophage (n = 20, 10.3%); no significant attenuation (n = 13, 6.7%) (Fig. 3). The intra- and inter-observer variability were 0.95 and 0.81, respectively. To assess the impact of plaque burden on the concordance between RF-IVUS and OCT, we evaluated the concordance rate among sub-groups stratified according to the mean plaque burden of the lesion (Supplementary Table 1). The concordance rate for TCFA diagnosis was 31.2% (RF-TCFA and OCT-TCFA: 0.7%, RF-non-TCFA and OCT-non-TCFA: 30.4%) in lesions with mean plaque burden ≥ 40% and < 50% (n = 138), and 24.6% (RF-TCFA and OCT-TCFA: 10.3%, RF-non-TCFA and OCT-non-TCFA: 14.3%) in lesions with mean plaque burden ≥ 50% (n = 126). When applying the definition of OCT-TCFA with minimum FCT < 75 μm, the concordance rate between RF-IVUS and OCT for TCFA diagnosis increased to 34.4% (RF-TCFA and OCT-TCFA: 9.8%, RF-non-TCFA and OCT-non-TCFA: 24.6%) (Supplementary Table 2).

### Distribution of minimal fibrous cap thickness of OCT-FA among RF-TCFA

**Fig. 4:**
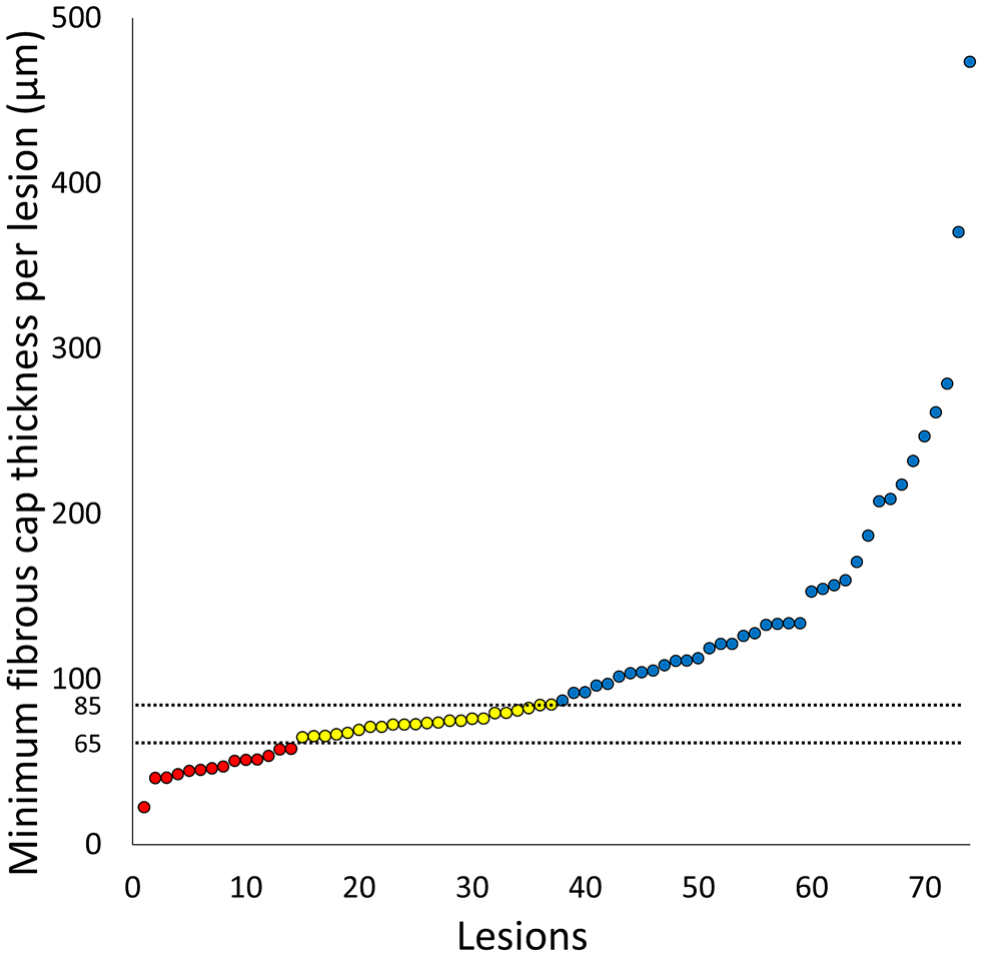
Distribution of minimal fibrous cap thickness of OCT-FA lesions among RF-TCFA lesions (n = 74). *FA* fibroatheroma, *OCT* optical coherence tomography, *TCFA* thin-cap fibroatheroma, *RF* radiofrequency

Figure 4 summarizes the distribution of minimal FCT of OCT-FA among RF-TCFA lesions. FCT ranged from 23 to 473 µm, with a median of 86 µm (interquartile range 65 µm). Among 74 RF-TCFA with OCT-FA morphology, 18.9% had minimum FCT ≤ 65 µm and 50.0% had minimum FCT ≤ 85 µm by OCT.

### IVUS and OCT analysis according to the concordance between RF- and OCT-TCFA

Table 3 summarizes quantitative IVUS and OCT analysis according to the concordance between RF- and OCT-TCFA. Lesions with RF- and OCT-TCFA had smaller minimum lumen area, greater percent atheroma volume, greater remodeling index, and greater percent necrotic core area compared with other groups.

## Discussion

The major findings of the present study were (1) there was a substantial discordance (70.3%) in the assessment of TCFA between RF-IVUS and OCT. The vast majority of RF-TCFA were not categorized as TCFA by OCT, while all OCT-TCFA were classified as TCFA by RF-IVUS, and (2) the reasons for discordance between RF-TCFA and OCT-non-TCFA were OCT-fibrous plaque (34%), OCT-ThCFA (26%), attenuation due to calcium (23%), attenuation due to macrophage accumulations (10%), and no significant attenuation (7%).

Although previous ex-vivo studies have demonstrated that both RF-IVUS and OCT had a good diagnostic accuracy (76−98%) for TCFA compared with histology [9, 18, 19], there is limited data on the direct comparison of TCFA detection between RF-IVUS and OCT. Previous in-vivo imaging studies have reported a lower discordance rate between RF-IVUS and OCT for in-vivo TCFA identification (≈ 20–30%) compared with the present study; however, underlying reasons for the discrepancy remain poorly understood [20, 21].

There are several explanations for the disagreement between RF-IVUS and OCT for the diagnosis of TCFA. First, the commonly acknowledged cut-off of fibrous cap thickness threshold to define TCFA is 65 µm, which is below the resolution of IVUS (100–150 µm). Unlike OCT, RF-IVUS defines TCFA as “ > 10% confluent NC abutting to the lumen for > 30° in ≥ 3 consecutive frames” without consideration of cap thickness, which was based on the consensus among experts rather than the result of an autopsy validation study [3]. This inherent limitation of RF-IVUS may result in an overestimation of the incidence of TCFA as observed in the current study. OCT is considered the gold standard diagnostic tool to correctly measure the fibrous cap thickness in vivo; as reportedly shown in a previous histology study suggesting a good correlation of the fibrous cap thickness between OCT and histology (r = 0.90, P < 0.001) [22].

Since the definition of RF-TCFA entirely depends on the amount and location of NC displayed by the decision algorithm, an overestimation of necrotic core by RF-IVUS may have resulted in a notable false-positive rate of RF-TCFA identification compared with OCT. A previous study comparing histology with RF-IVUS and OCT showed that the misclassification of necrotic core was commonly encountered in the region of calcifications, in which the adjacent plaque composition is incorrectly categorized as NC [18]. The misclassification is potentially related to calcium being included in the algorithm for the detection of necrotic core [2]. In our study, this was the case in 23% of RF-IVUS TCFA lesions. Although the detection of necrotic core behind a superficial calcific pool might be impossible by OCT [23], such a plaque by definition does not fulfill the definition of a TCFA (i.e. thin fibrous cap overlying necrotic core). With regards to fibrous plaques, an ex-vivo animal study comparing RF-IVUS vs. histology reported that dense fibrous tissue was falsely categorized as necrotic core [24]. In accordance with this data, our study revealed that 34% of plaques with a fibrous morphology by OCT were categorized as TCFA by RF-IVUS.

Some technical aspects of RF-IVUS also deserve consideration. First, RF-IVUS catheters used in the IBIS-4 study (i.e. s5) may overestimate the amount of NC compared with its precursor (i.e. in-vision gold). Second, the correct diagnosis of RF-TCFA especially in cross-sections with moderate plaque burden might be more challenging, although a sensitivity analysis did not show a relevant difference in the concordance rate as a function of plaque burden (Supplementary Table 1). Of note, and in line with our findings, a higher prevalence of RF-TCFA has been reported in clinical studies investigating non-flow-limiting lesions (21.6% in PROSPECT [5], 41% in HORIZON AMI [25], 37.4% in ATHEROREMO-IVUS [6], 61.6% in VIVA [7]) compared with the OCT-TCFA (18% in Tian et al. [26], 15.6% in IBIS-4 [27], 7.3% in Tanaka et al. [28]) and histology-defined TCFA of 10.8% [29], suggesting a relatively consistent overestimation in TCFA diagnosis by RF-IVUS in the literature. Furthermore, the GLAGOV RF-IVUS sub-analysis has recently demonstrated that RF-IVUS was unable to detect the significant regression in necrotic core between the evolocumab and placebo groups (Δ 76 weeks—baseline: − 0.6 ± 0.5 mm^3^ vs. − 0.1 ± 0.5 mm^3^, P = 0.49) despite the significant atheroma regression in the evolocumab group, questioning the utility of RF-IVUS in assessing the effects of antiatherosclerotic therapies [30].

Although all OCT-TFCA were confirmed as TCFA by RF-IVUS in the current study, the presence of macrophages, calcifications, and artefacts render TCFA diagnosis by OCT challenging as suggested by several ex vivo studies [31, 32]. A previous OCT study demonstrated that standardization of in-vivo FCT assessment by OCT and development of image interpretation criteria resulted in significantly higher levels of interobserver variability of FCT measurement [33].

From a clinical perspective, the PROSPECT and VIVA have demonstrated that RF-TCFA was associated with subsequent major adverse events [5, 7]. Given the overestimation of RF-TCFA compared with OCT-TCFA as shown in the present study, the prognostic value of RF-TCFA shown in the PROSPECT and VIVA appeared due to the amount of NC and co-existing other features such as plaque burden and lumen area rather than fibrous cap thickness itself. At variance with the PROSPECT and VIVA, in the ATHEROREMO-IVUS study, the presence of RF-TCFA with a plaque burden ≥ 70% was significantly associated with MACE (cumulative MACE incidence when present: 37.7% vs. 24.6% when absent; adjusted HR: 1.73; 95% CI 1.12 to 2.66; P = 0.013), while the presence of a TCFA lesion alone was not independently associated with MACE [34]. Although differences in study design across trials need to be considered, an incremental value of RF-derived plaque characterization over grayscale IVUS (i.e. plaque burden assessment) remained uncertain. The prognostic significance of OCT-TCFA has been demonstrated in a few prospective studies (the CLIMA [35] and the COMBINE OCT-FFR study [36]). Direct comparison of the prognostic significance between RF- vs. OCT-TCFA is not available to date. Near-infrared spectroscopy (NIRS) has recently become available as an alternative method to identify the extent of lipid-rich plaque in the coronary arteries. The LRP study and PROSPECT II study have reported that lipid core burden index as assessed by NIRS was significantly associated with future MACE [37]. Since RF-IVUS is no longer used in the daily practice, OCT and NIRS are the preferred methods to detect the vulnerable plaque, although no adequately powered prospective randomized trials assessing the impact of vulnerable plaque detection by intracoronary imaging on the subsequent treatment strategy have been conducted to date.

### Limitations

Several limitations should be considered in the present study. First, only STEMI patients were enrolled in the IBIS-4 study, which may limit the generalizability of the findings to other patient subsets (i.e. chronic coronary syndrome). Second, although matching between RF-IVUS and OCT was performed using a dedicated matching software by expert analysts, small longitudinal mismatches may affect the findings of the current study. Third, the findings of the current study do not apply to other radiofrequency analysis methods for plaque classification. Fourth, although the current study provides the important insights for better understanding of vulnerable plaque features defined by RF-IVUS and OCT, the clinical significance of the current study may be limited since RF-IVUS is no longer used in the daily practice.

## Conclusions

There was a notable discordance in the diagnostic assessment of TCFA between RF-IVUS and OCT. The majority of RF-IVUS derived TCFA were not categorized as fibroatheroma using OCT, while all OCT derived TCFA were classified as fibroatheroma by RF-IVUS.

## Supplementary Information

Below is the link to the electronic supplementary material.

Supplementary file1 (DOCX 18 kb)

## References

[1] Virmani R, Burke AP, Farb A, Kolodgie FD (2006). Pathology of the vulnerable plaque. J Am Coll Cardiol.

[2] Nair A, Kuban BD, Obuchowski N, Vince DG (2001). Assessing spectral algorithms to predict atherosclerotic plaque composition with normalized and raw intravascular ultrasound data. Ultrasound Med Biol.

[3] Garcia-Garcia HM, Mintz GS, Lerman A, Vince DG, Margolis MP, van Es GA (2009). Tissue characterisation using intravascular radiofrequency data analysis: recommendations for acquisition, analysis, interpretation and reporting. EuroIntervention.

[4] Tearney GJ, Regar E, Akasaka T, Adriaenssens T, Barlis P, Bezerra HG (2012). Consensus standards for acquisition, measurement, and reporting of intravascular optical coherence tomography studies: a report from the International Working Group for Intravascular Optical Coherence Tomography Standardization and Validation. J Am Coll Cardiol.

[5] Stone GW, Maehara A, Lansky AJ, de Bruyne B, Cristea E, Mintz GS (2011). A prospective natural-history study of coronary atherosclerosis. N Engl J Med.

[6] Cheng JM, Garcia-Garcia HM, de Boer SP, Kardys I, Heo JH, Akkerhuis KM (2014). In vivo detection of high-risk coronary plaques by radiofrequency intravascular ultrasound and cardiovascular outcome: results of the ATHEROREMO-IVUS study. Eur Heart J.

[7] Calvert PA, Obaid DR, O'Sullivan M, Shapiro LM, McNab D, Densem CG (2011). Association between IVUS findings and adverse outcomes in patients with coronary artery disease: the VIVA (VH-IVUS in Vulnerable Atherosclerosis) Study. JACC Cardiovasc Imaging.

[8] Xing L, Higuma T, Wang Z, Aguirre AD, Mizuno K, Takano M (2017). Clinical significance of lipid-rich plaque detected by optical coherence tomography: a 4-year follow-up study. J Am Coll Cardiol.

[9] Obaid DR, Calvert PA, Gopalan D, Parker RA, Hoole SP, West NE (2013). Atherosclerotic plaque composition and classification identified by coronary computed tomography: assessment of computed tomography-generated plaque maps compared with virtual histology intravascular ultrasound and histology. Circ Cardiovasc Imaging.

[10] Nair A, Margolis MP, Kuban BD, Vince DG (2007). Automated coronary plaque characterisation with intravascular ultrasound backscatter: ex vivo validation. EuroIntervention.

[11] Yabushita H (2002). Characterization of human atherosclerosis by optical coherence tomography. Circulation.

[12] Raber L, Kelbaek H, Ostoijc M, Baumbach A, Tuller D, von Birgelen C (2012). Comparison of biolimus eluted from an erodible stent coating with bare metal stents in acute ST-elevation myocardial infarction (COMFORTABLE AMI trial): rationale and design. EuroIntervention.

[13] Raber L, Kelbaek H, Ostojic M, Baumbach A, Heg D, Tuller D (2012). Effect of biolimus-eluting stents with biodegradable polymer vs bare-metal stents on cardiovascular events among patients with acute myocardial infarction: the COMFORTABLE AMI randomized trial. JAMA.

[14] Raber L, Taniwaki M, Zaugg S, Kelbaek H, Roffi M, Holmvang L (2015). Effect of high-intensity statin therapy on atherosclerosis in non-infarct-related coronary arteries (IBIS-4): a serial intravascular ultrasonography study. Eur Heart J.

[15] Raber L, Mintz GS, Koskinas KC, Johnson TW, Holm NR, Onuma Y, et al. Clinical use of intracoronary imaging. Part 1: guidance and optimization of coronary interventions. An expert consensus document of the European Association of Percutaneous Cardiovascular Interventions: Endorsed by the Chinese Society of Cardiology. *Eur Heart J* 2018. 10.1093/eurheartj/ehy285 29790954

[16] Maehara A, Cristea E, Mintz GS, Lansky AJ, Dressler O, Biro S (2012). Definitions and methodology for the grayscale and radiofrequency intravascular ultrasound and coronary angiographic analyses. JACC Cardiovasc Imaging.

[17] Mintz GS, Garcia-Garcia HM, Nicholls SJ, Weissman NJ, Bruining N, Crowe T (2011). Clinical expert consensus document on standards for acquisition, measurement and reporting of intravascular ultrasound regression/progression studies. EuroIntervention.

[18] Brown AJ, Obaid DR, Costopoulos C, Parker RA, Calvert PA, Teng Z (2015). Direct comparison of virtual-histology intravascular ultrasound and optical coherence tomography imaging for identification of thin-cap fibroatheroma. Circ Cardiovasc Imaging.

[19] Kume T, Okura H, Yamada R, Kawamoto T, Watanabe N, Neishi Y (2009). Frequency and spatial distribution of thin-cap fibroatheroma assessed by 3-vessel intravascular ultrasound and optical coherence tomography: an ex vivo validation and an initial in vivo feasibility study. Circ J.

[20] Sawada T, Shite J, Garcia-Garcia HM, Shinke T, Watanabe S, Otake H (2008). Feasibility of combined use of intravascular ultrasound radiofrequency data analysis and optical coherence tomography for detecting thin-cap fibroatheroma. Eur Heart J.

[21] Kubo T, Nakamura N, Matsuo Y, Okumoto Y, Wu X, Choi SY (2011). Virtual histology intravascular ultrasound compared with optical coherence tomography for identification of thin-cap fibroatheroma. Int Heart J.

[22] Kume T, Akasaka T, Kawamoto T, Okura H, Watanabe N, Toyota E (2006). Measurement of the thickness of the fibrous cap by optical coherence tomography. Am Heart J.

[23] Di Vito L, Imola F, Gatto L, Romagnoli E, Limbruno U, Marco V (2017). Limitations of OCT in identifying and quantifying lipid components: an in vivo comparison study with IVUS-NIRS. EuroIntervention.

[24] Thim T, Hagensen MK, Wallace-Bradley D, Granada JF, Kaluza GL, Drouet L (2010). Unreliable assessment of necrotic core by virtual histology intravascular ultrasound in porcine coronary artery disease. Circ Cardiovasc Imaging.

[25] Zhao Z, Witzenbichler B, Mintz GS, Jaster M, Choi SY, Wu X (2013). Dynamic nature of nonculprit coronary artery lesion morphology in STEMI: a serial IVUS analysis from the HORIZONS-AMI trial. JACC Cardiovasc Imaging.

[26] Tian J, Dauerman H, Toma C, Samady H, Itoh T, Kuramitsu S (2014). Prevalence and characteristics of TCFA and degree of coronary artery stenosis: an OCT, IVUS, and angiographic study. J Am Coll Cardiol.

[27] Raber L, Koskinas KC, Yamaji K, Taniwaki M, Roffi M, Holmvang L, et al. Changes in coronary plaque composition in patients with acute myocardial infarction treated with high-intensity statin therapy (IBIS-4): a serial optical coherence tomography study. JACC Cardiovasc Imaging 2018. 10.1016/j.jcmg.2018.08.024 30553686

[28] Tanaka A, Imanishi T, Kitabata H, Kubo T, Takarada S, Kataiwa H et al. Distribution and frequency of thin-capped fibroatheromas and ruptured plaques in the entire culprit coronary artery in patients with acute coronary syndrome as determined by optical coherence tomography. *Am J Cardiol* 2008;8;975–979.29. 10.1016/j.amjcard.2008.05.062 18929696

[29] Cheruvu PK, Finn AV, Gardner C, Caplan J, Goldstein J, Stone GW (2007). Frequency and distribution of thin-cap fibroatheroma and ruptured plaques in human coronary arteries: a pathologic study. J Am Coll Cardiol.

[30] Nicholls SJ, Puri R, Anderson T, Ballantyne CM, Cho L, Kastelein JJP (2018). Effect of Evolocumab on Coronary Plaque Composition. J Am Coll Cardiol.

[31] Manfrini O, Mont E, Leone O, Arbustini E, Eusebi V, Virmani R (2006). Sources of error and interpretation of plaque morphology by optical coherence tomography. Am J Cardiol.

[32] van Soest G, Regar E, Goderie TP, Gonzalo N, Koljenovic S, van Leenders GJ (2011). Pitfalls in plaque characterization by OCT: image artifacts in native coronary arteries. JACC Cardiovasc Imaging.

[33] Kini AS, Vengrenyuk Y, Yoshimura T, Matsumura M, Pena J, Baber U, et al. Assessment of Fibrous Cap Thickness by Optical Coherence Tomography In Vivo: Reproducibility and Standardization. J Am Coll Cardiol 2016. 10.1016/j.jacc.2016.10.028 27989887

[34] Schuurman AS, Vroegindewey MM, Kardys I, Oemrawsingh RM, Garcia-Garcia HM, van Geuns RJ (2018). Prognostic value of intravascular ultrasound in patients with coronary artery disease. J Am Coll Cardiol.

[35] Prati F, Romagnoli E, Gatto L, La Manna A, Burzotta F, Ozaki Y (2020). Relationship between coronary plaque morphology of the left anterior descending artery and 12 months clinical outcome: the CLIMA study. Eur Heart J.

[36] Kennedy MW, Fabris E, Ijsselmuiden AJ, Nef H, Reith S, Escaned J (2016). Combined optical coherence tomography morphologic and fractional flow reserve hemodynamic assessment of non- culprit lesions to better predict adverse event outcomes in diabetes mellitus patients: COMBINE (OCT-FFR) prospective study. Rationale Design Cardiovasc Diabetol.

[37] Waksman R, Di Mario C, Torguson R, Ali ZA, Singh V, Skinner WH (2019). Identification of patients and plaques vulnerable to future coronary events with near-infrared spectroscopy intravascular ultrasound imaging: a prospective, cohort study. Lancet.

