# Supplementary material for: Discordance in the diagnostic assessment of vulnerable plaques between radiofrequency intravascular ultrasound versus optical coherence tomography among patients with acute myocardial infarction: insights from the IBIS-4 study
Source: Int J Cardiovasc Imaging. 2021 Jul 8;37(10):2839–47. doi: 10.1007/s10554-021-02272-6 (PMC8494667; doi:10.1007/s10554-021-02272-6)
Supplement: Supplementary file 1 — Supplementary file1 (DOCX 18 kb) [file 10554_2021_2272_MOESM1_ESM.docx]

**Supplementary Table 1.** Plaque classification according to plaque burden

| **Lesions with mean plaque burden ≥40%, <50% (n=138)** | | | | | |
| --- | --- | --- | --- | --- | --- |
| **RF-IVUS** | **OCT** | | | | |
|  | TCFA  (n=1) | ThCFA  (n=36) | Fibrocalcific plaque  (n=63) | Fibrous plaque  (n=28) | Normal vessel  (n=10) |
| TCFA (n=96) | 1 | 26 | 49 | 16 | 4 |
| ThCFA (n=20) | 0 | 5 | 9 | 5 | 1 |
| Fibrocalcific (n=3) | 0 | 0 | 3 | 0 | 0 |
| Fibrotic (n=7) | 0 | 2 | 1 | 2 | 2 |
| PIT (n=12) | 0 | 3 | 1 | 5 | 3 |
| **Lesions with mean plaque burden ≥50% (n=126)** | | | | | |
| **RF-IVUS** | **OCT** | | | | |
|  | TCFA  (n=13) | ThCFA  (n=39) | Fibrocalcific plaque  (n=60) | Fibrous plaque  (n=14) | Normal vessel  (n=0) |
| TCFA (n=96) | 13 | 32 | 52 | 11 | 0 |
| ThCFA (n=17) | 0 | 7 | 8 | 2 | 0 |
| Fibrocalcific (n=0) | 0 | 0 | 0 | 0 | 0 |
| Fibrotic (n=1) | 0 | 0 | 0 | 1 | 0 |
| PIT (n=0) | 0 | 0 | 0 | 0 | 0 |

IVUS = intravascular ultrasound, OCT = optical coherence tomography, PIT = pathological intimal thickening, TCFA = thin-cap fibroatheroma, ThCFA = thick-cap fibroatheroma, RF = radiofrequency.

**Supplementary Table 2.** Plaque classification as assessed by RF-IVUS and OCT with definition of OCT-TCFA with minimum FCT <75μm

| Lesions (n=276) |  | | | | |
| --- | --- | --- | --- | --- | --- |
| **RF-IVUS** | **OCT** | | | | |
|  | TCFA  (n=14) | ThCFA  (n=77) | Fibrocalcific plaque  (n=126) | Fibrous plaque  (n=47) | Normal vessel  (n=12) |
| TCFA (n=208) | 27 | 47 | 101 | 29 | 4 |
| ThCFA (n=39) | 0 | 12 | 19 | 7 | 1 |
| Fibrocalcific (n=3) | 0 | 0 | 3 | 0 | 0 |
| Fibrotic (n=9) | 0 | 2 | 1 | 4 | 2 |
| PIT (n=17) | 0 | 3 | 2 | 7 | 8 |

IVUS = intravascular ultrasound, OCT = optical coherence tomography, PIT = pathological intimal thickening, TCFA = thin-cap fibroatheroma, ThCFA = thick-cap fibroatheroma, RF = radiofrequency.
